# Immunological characterization of an Italian PANDAS cohort

**DOI:** 10.3389/fped.2023.1216282

**Published:** 2024-01-04

**Authors:** Lucia Leonardi, Giulia Lorenzetti, Rita Carsetti, Eva Piano Mortari, Cristiana Alessia Guido, Anna Maria Zicari, Elisabeth Förster-Waldl, Lorenzo Loffredo, Marzia Duse, Alberto Spalice

**Affiliations:** ^1^ Department of Maternal, Infantile and Urological Sciences, Sapienza University of Rome, Rome, Italy; ^2^ Department of Pediatrics, University of Rome Tor Vergata, Rome, Italy; ^3^ B Cell Physiopathology Unit, Immunology Research Area, Bambino Gesù Children Hospital, Rome, Italy; ^4^ Division of Neonatology, Pediatric Intensive Care and Neuropediatrics, Department of Pediatrics & Adolescent Medicine, Center for Congenital Immunodeficiencies, Medical University of Vienna, Vienna, Austria; ^5^ Department of Clinical, Internal Medicine, Anesthesiologic and Cardiovascular Sciences, Sapienza University of Rome, Rome, Italy

**Keywords:** PANDAS, PANS, GABHS, TNF-α, IL-17, immune defects, neuropsychiatric symptoms

## Abstract

This cross-sectional study aimed to contribute to the definition of Pediatric Autoimmune Neuropsychiatric Disorders Associated with Streptococcal Infections (PANDAS) pathophysiology. An extensive immunological assessment has been conducted to investigate both immune defects, potentially leading to recurrent Group A β-hemolytic Streptococcus (GABHS) infections, and immune dysregulation responsible for a systemic inflammatory state. Twenty-six PANDAS patients with relapsing-remitting course of disease and 11 controls with recurrent pharyngotonsillitis were enrolled. Each subject underwent a detailed phenotypic and immunological assessment including cytokine profile. A possible correlation of immunological parameters with clinical-anamnestic data was analyzed. No inborn errors of immunity were detected in either group, using first level immunological assessments. However, a trend toward higher TNF-alpha and IL-17 levels, and lower C3 levels, was detected in the PANDAS patients compared to the control group. Maternal autoimmune diseases were described in 53.3% of PANDAS patients and neuropsychiatric symptoms other than OCD and tics were detected in 76.9% patients. ASO titer did not differ significantly between the two groups. A possible correlation between enduring inflammation (elevated serum TNF-α and IL-17) and the persistence of neuropsychiatric symptoms in PANDAS patients beyond infectious episodes needs to be addressed. Further studies with larger cohorts would be pivotal to better define the role of TNF-α and IL-17 in PANDAS pathophysiology.

## 1 Introduction

*Streptococcus pyogenes* [Group A β-hemolytic Streptococcus (GABHS)] is responsible for a broad spectrum of infectious and autoimmune diseases resulting in high morbidity, being the primary agent for acute pharyngitis in children worldwide. Indeed, more than 600 million cases of symptomatic GABHS pharyngitis occur annually worldwide among children older than 4 years of age (1). GABHS related disease can result from direct infection, toxin production or immune mediated non suppurative processes, including autoimmune sequelae like rheumatic fever (RF), Sydenham's chorea (SC) and glomerulonephritis. The spectrum of post-streptococcal immune mediated neurological diseases includes movement disorders (chorea, tics, dystonia and parkinsonism) and psychiatric disorders, such as obsessive-compulsive disorder (OCD) (2). However, it is remarkable how, despite its extremely high incidence in pediatric age, only a minority of children show neuropsychiatric symptoms following GABHS infection. This evidence suggests that the onset of central nervous system (CNS) autoimmune symptoms is the result of a multifactorial process, which might be influenced by genetic susceptibility to specific GABHS strains (3). In 1998, Swedo and colleagues described a cohort of 50 pediatric patients with acute onset of OCD and/or tics associated with other neurological and psychiatric abnormalities (4). Symptom onset was clinically related to previous and repeated exposures to GABHS. On this occasion the acronym PANDAS (Pediatric Autoimmune Neuropsychiatric Disorders Associated with Streptococcal Infections) was proposed. In the past 20 years, there has been a growing interest in the association between infections, autoimmunity, and behavioral changes in PANDAS patients, together with an increased awareness and interest in the brain-immunity axis. Because of many immunological and clinical similarities between PANDAS and SC, the latter has been the practical-theoretical model for interpreting PANDAS pathophysiology. Both conditions are considered to be the result of post-infectious autoimmunity mediated by antibodies produced against GABHS which cross-react with neuronal targets in the basal ganglia in predisposed patients, following a phenomenon of molecular mimicry (5). However, despite both SC and PANDAS are characterized by an abrupt onset of the disease, SC is most-often a monophasic illness, while the recurrence of tics or OCD symptoms represents a major criterion for PANDAS diagnosis. Recently, Wald et al. reported an estimated annual incidence of PANDAS/PANS at 1 in 11,765 for children aged 3–12 years, with some variation observed among different geographic areas (6). Data on the exact prevalence in Italy are still unknown. To date, PANDAS diagnosis is based on a clinical evaluation and defined by specific criteria (4), while an open debate regarding its etiology and its specific pathophysiology is ongoing. A critical aspect of PANDAS diagnosis is that studies conducted so far showed conflicting data about the causal association with GABHS or possible immunological alterations that might predispose to recurrent GABHS infections (7). Therefore, different clinical conditions such as “Pediatric Acute-onset Neuropsychiatric Syndrome” (PANS), “Childhood Acute Neuropsychiatric Syndrome” (CANS) or “Pediatric Infections-Triggered Autoimmune Neuropsychiatric disorders” (PITANDs) have been defined to overcome the frequent difficulty in correlating neuropsychiatric symptoms onset to GABHS infection, allowing other possible etiological agents to be considered. Nevertheless, some authors consider PANDAS as a subtype of PANS (8–10) and the causal association with GABHS is supported by independent preclinical studies. Dileepan et al. demonstrated in murine models the pathogenic link between repeated intranasal exposures to GABHS and neurobehavioral abnormalities via autoantibodies production targeting specific brain areas after the disruption of the blood-brain barrier (BBB) (11, 12). Indeed, it has been hypothesized that specific subtype of lymphocytes, T helper 17 (Th17) and Th17 derived cytokines [including interleukin (IL)-17] are involved in BBB leakage allowing the cross reaction of antistreptococcal antibodies to brain targets located in the basal ganglia through a process of molecular mimicry (11–13). Basal ganglia are involved in motor, cognitive and emotional functions, being implicated in the pathogenesis of OCD and tics. Motor abnormalities observed in PANDAS patients have been related to higher titers of anti-basal ganglia antibodies compared to healthy controls (14, 15). Although emerging data is revealing the mechanisms by which GABHS-specific Th17 cells and antineuronal antibodies cross the BB and enter the brain, additional work needs to be done (16). Th17 cells have also been implicated in the pathogenesis of other autoimmune neurological diseases, including multiple sclerosis (MS), where they trigger inflammation, BBB destruction and tissue damage. IL-17 is known to be able to disrupt BBB integrity *in vivo* and *in vitro* through the production of reactive oxygen species (ROS) in human brain-derived endothelial cells (17–19). Moreover, IL-17 concentration in the cerebrospinal fluid (CSF) of MS patients correlates with impairment of BBB integrity, suggesting a direct contribution of this cytokine in MS pathophysiology (20). To date, several therapeutic options have been empirically used in PANDAS patients, mostly including antibiotic therapy (or prophylaxis), with limited clinical outcome in most patients. On the contrary, alternative treatment including immunomodulatory strategies [i.e., intravenous immunoglobulin (IVIG)] still needs to be better investigated. In this perspective, a critical aspect of PANDAS management is the lack of reliable biological markers. Improving our knowledge of PANDAS pathogenesis would offer better chances to treat the extremely disabling neurological and psychiatric symptoms impairing PANDAS children's quality of life. The aim of our study was to provide a contribution to the definition of PANDAS pathophysiology, potentially able to suggest disease-associated biomarkers, thereby improving therapeutic options. The primary objective was to conduct an immunological evaluation in PANDAS patients in order to investigate possible immunological defects compatible with the susceptibility to recurrent GABHS infections. The secondary objective was to verify the hypothesis that PANDAS symptoms could result from a persistent inflammatory status more than a recurrence of infections. We analyzed cytokine profile and its possible correlation with clinical phenotype, anamnestic data, laboratory findings and neuropsychological tests score to investigate possible immune dysregulation leading to systemic inflammatory state.

## 2 Materials and methods

### 2.1 Population

Our cross-sectional study was conducted on a cohort of 26 patients (males = 20, females = 6, aged 3–15 years old), including two pairs of siblings with a clinical diagnosis of PANDAS as defined by Swedo et al. (4). The control group (*N* = 11, males = 5, females = 6) consisted of children (3–15 years old) with a history of recurrent GAS pharyngotonsillitis, being GAS negative at the time of the study. Children in the control group were also screened to exclude any neuropsychiatric disorder. All subjects were enrolled at the Pediatric Department of Umberto I Hospital in Rome, Italy. Prior informed consent obtained by both parents and approved by the local Ethics Committee, general and neurological examination, routine blood tests [complete blood count (CBC), erythrocyte sedimentation rate (ESR), C-reactive protein (CRP), hepatic and renal function, thyroid function, electrolytes], anti-streptolysin O (ASO) titer and pharyngeal swab were performed for each subject. Anamnestic data collected included: family history (first- and second-degree relatives), age at symptoms onset, number of flares in the previous year and related symptoms, potential triggers and course of disease, culture-verified infections, previous and current medication including number of treatment courses. The immunological evaluation included serum immunoglobulin (Ig) levels, IgG subclasses, lymphocyte subsets count, vaccine response (anti-haemophilus, anti-pneumococcal and anti-tetanus antibody titers), complement analysis (C3, C4), antinuclear antibodies (ANA), anti-double stranded DNA (anti-dsDNA) antibodies, antibodies to Extractable Nuclear Antigens (anti-ENA antibodies). Additionally, cytokine profiling [IL-17, IL-1β, IL-6, tumor necrosis factor-alpha (TNF-α)] was performed in all subjects. Cytokine quantification was assessed by bead-based ELISA multiplex, using a Milliplex Human Cytokine Magnetic bead customized panel, and following the manufacturer's instruction (Millipore, Chicago, IL). Analysis was performed at the Luminex® 100/200 ™ System with xPOTENT® software (Luminex, Austin, TX).

Tic severity was rated on the Yale Global Tic Severity Score (YGTSS) on 23 patients (>6 years of age) (21). OCD severity was rated on the Children's Yale-Brown Obsessive-Compulsive Scale (CY-BOCS) on 23 patients (>6 years of age) (22). These tests are routinely employed in both clinical practice and clinical trials, being the gold standard instruments to quantify the severity of OCD and tic disorder symptoms.

### 2.2 Statistical analysis

Data are described as mean with standard deviation (SD) or median with interquartile range (IQR) for continuous variables. A nonparametric analysis (Mann–Whitney *U*-test) for continuous variables and the chi-square or Fisher's exact test for categorical variables were used to measure differences between groups. Pearson correlation coefficients were calculated to test for possible associations among variables. Statistical significance for all analyses was set at the level of.05 for a two-tailed test. Stata 14.2 program and SPSS 15.0 software were used for all statistical computations.

## 3 Results

A total of 26 PANDAS patients [20 males (76.9%)] were enrolled. The age at symptoms onset ranged from 2 to 15 years (mean ± SD: 6 ± 2.9). Median (IQR) time since symptoms onset was 2 years (0–8). All patients presented with a relapsing-remitting course of disease. Family history was positive for maternal autoimmunity in 14/24 (53.3%) cases: 12 cases of autoimmune thyroiditis, 1 psoriasis, 1 Sjogren syndrome. Three (11.5%) patients had a family history positive for rheumatic fever, and 1 (3.8%) patient for OCD. Clinical manifestations in the previous year were characterized by motor or vocal tics in 11 (42.3%) subjects, while 3 (11.5%) patients presented with OCD exclusively and 12 patients (46.1%) presented with OCD + tics. Twenty (76.9%) patients reported neuropsychiatric symptoms other than OCD and tics [i.e., emotional lability, depression, irritability, aggression, oppositional behaviors, behavioral developmental regression, deterioration in school performance (mostly in handwriting), separation anxiety and sleep disorder]. In the year before our evaluation patients were mostly treated with antibiotics [penicillin in 8/26 patients (30.8%), amoxicillin/clavulanate in 19/26 patients (73.1%), azithromycin in 7/26 patients (26.9%)] and cognitive-behavioral therapy in 3/26 patients (11.5%). None of our patients were prescribed steroids or intravenous immunoglobulin. Only three patients (11.5%) underwent tonsillectomy. At the moment of our evaluation, none of the patients was taking medications (e.g., antibiotics, anti-histamines, NSAIDS, steroids or psychotropic drugs). Two (7.7%) patients showed motor tics during our clinical examination. Tic severity, rated on the YGTSS on 23 patients, showed a mean value of 39.2 ± 11.8 (Global Severity Score range: 0–100). OCD severity, rated on the CY-BOCS on 23 patients, showed a mean value of 18.2 ± 5.3 [subclinical (0–7), mild (8–15), moderate (16–23), severe (24–31), extreme (32–40)]. The previous score was not available for both CY-BOCS and YGTSS. Pharyngeal swab for GABHS detection was performed in all patients and controls resulting in negative culture in all subjects. On the contrary, ASO titer resulted to be higher than the reference value of our laboratory (i.e., >166 IU/ml) in 24 patients (92.3%), ranging from 245 to 1,427 IU/ml, with a median (IQR) of 303.5 IU/ml (213–422). In the control group, ASO titer was elevated in 8/9 (88.8%) subjects, ranging from 68 IU/ml to 1,335 IU/ml with a median (IQR) of 407 IU/ml (311–1,007). ASO titer value was not significantly different between the two groups (Table 1). No patient presented with acute pharyngotonsillitis. CRP was negative in all patients, while ESR was positive only in 2/26 (7.7%) patients.

**Table 1:** Immunological work-up.

	Controls (*N* = 11)		Patients (*N* = 26)		
	Median	IQR	Median	IQR	*p*
WBCs (×10^9 ^/L)	6.52	5.4–7.1	5.88	5.0–7.5	0.595
Neutrophils (×10^9^ /L)	2.67	2.5–2.9	2.56	2.2–3.2	0.506
Lymphocytes (×10^9^ /L)	3.07	2.2–3.5	2.45	2.2–3.2	0.344
	Controls (*N* = 11)		Patients (*N* = 26)		
	*N* out of range	%	*N* out of range	%	*p*
IgG	1	9.1	2	7.7	1.000
IgM	0	0.0	3	11.5	0.540
IgA	0	0.0	1	3.8	1.000
IgE	4	36.4	8	30.8	0.700
	Controls (*N* = 9)		Patients (*N* = 26)		
	*N* out of range	%	*N* out of range	%	*p*
IgG1	0	0.0	5	19.2	0.297
IgG2	1	11.1	0	0.0	0.257
IgG3	0	0.0	0	0.0	
IgG4	0	0.0	3	11.5	0.553
	Controls (*N* = 10)		Patients (*N* = 26)		
	*N* out of range	%	*N* out of range	%	*p*
CD3	2	20.0	2	7.7	0.305
CD4	4	40.0	3	11.5	0.086
CD8	3	30.0	4	15.4	0.370
CD56	1	10.0	2	7.7	1.000
CD19	0	0.0	4	15.4	0.550
	Controls (*N* = 10)		Patients (*N* = 26)		
	*N* out of range	%	*N* out of range	%	*p*
C3	0	0.0	8	30.8	0.076
C4	0	0.0	3	11.5	1.000
	Controls (*N* = 7)		Patients (*N* = 25)		
	*N* out of range	%	*N* out of range	%	*p*
Anti-tetanus Ab	1	14.3	0	0.0	0.219
Anti-pneumococcal Ab	1	14.3	5	20.0	1.000
Anti-haemophilus Ab	0	0.0	6	24.0	0.293
	Controls (*N* = 11)		Patients (*N* = 26)		
	*N* out of range	%	*N* out of range	%	*p*
ANA	0	0.0	0	0.0	
Anti-dsDNA	0	0.0	0	0.0	
Anti-ENA	0	0.0	0	0.0	
	Controls (*N* = 9)		Patients (*N* = 26)		*p*
ASO titer (UI/ml)					
*N* out of range, %	8	88.9	24	92.3	0.752
Median, IQR	407	311–1,007	303.5	213–422	0.186

Ab, antibodies; ANA, antinuclear antibodies; Anti-dsDNA, Anti-double stranded DNA, ASO, anti-streptolysin O; CD, cluster of differentiation; ENA, extractable nuclear antigen; Ig, immunoglobulin; IQR, interquartile range; *p*, *p*-value; WBCs, white blood cells.

### 3.1 Immunological work-up

**Figure 1:**
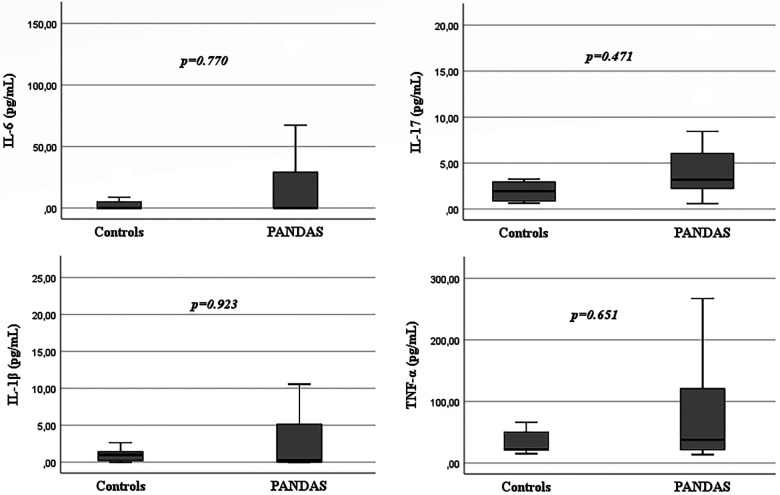
Cytokine profile: difference between PANDAS patients and controls.

**Figure 2:**
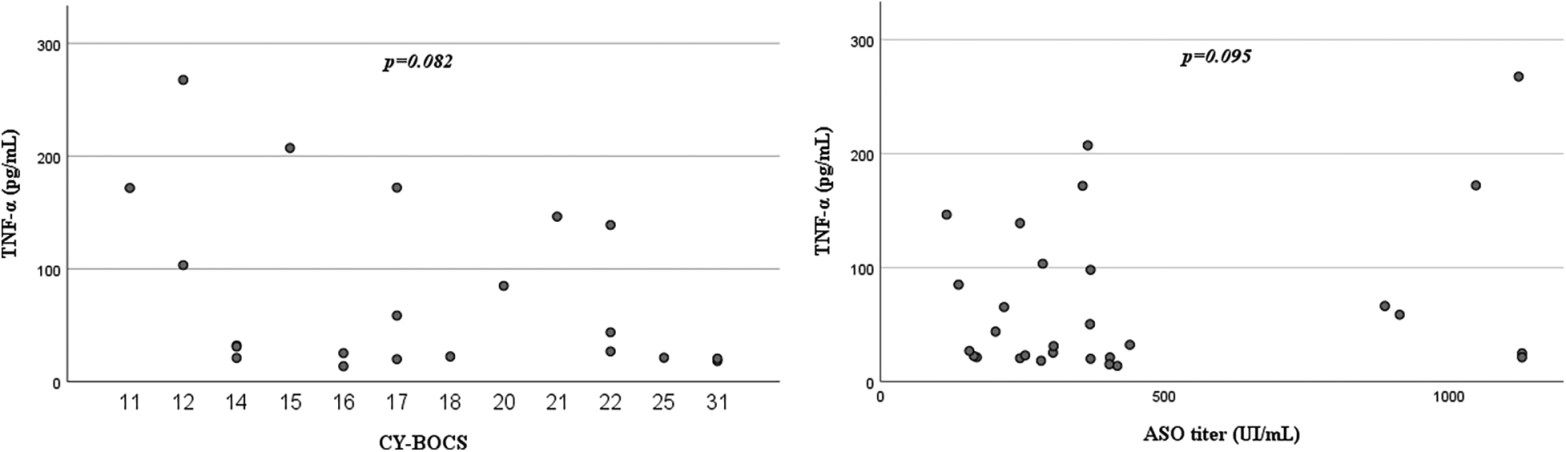
PANDAS patients: correlation between TNF-α and CY-BOCS; correlation between TNF-α and ASO titer.

CBC, lymphocyte subset analysis and serum IgG values were within the normal range for age in all patients. In the PANDAS group, 1 (3.8%) patient had incomplete IgA deficiency, 1 (3.8%) patient had low IgM, 8 (30.8%) patients had high serum IgE all related to allergic disease. Fifty percent (*N* = 4) of PANDAS patients with elevated total IgE levels had a positive family history of allergy but tested negative for specific IgE and had negative skin prick tests. Among the remaining four patients, two were receiving treatment for allergic asthma with inhalers, while the other two had allergic rhinoconjunctivitis. In the PANDAS population 8 (30.8%) patients had low C3 levels, 3 (11.5%) patients had low C4 levels, and 1 (3.8%) patient had both low C3 and C4 levels. Vaccine response was tested: all patients had protective anti-tetanus antibody titers, while 5/25 (20%) patients displayed antipneumococcal response considered to be only partially protective. ANA, anti-dsDNA and anti-ENA were negative in all patients. In contrast, none of the controls displayed any immunological alterations. Yet, no statistically significant difference was found between the two study groups in respect of immunological findings (Table 1). However, interestingly, 42.3% (11/26) of PANDAS patients showed complement alterations. More specifically, 30.8% of PANDAS patients showed reduced C3 levels when compared to controls (*p* = 0.076) and 11.5% of PANDAS patients showed reduced C4 levels (Table 1). By evaluation of cytokine profiles in serum, IL-1β-, IL-6-, IL-17- and TNF-α-concentrations were assessed. In the PANDAS group 2/24 (8.3%) patients showed elevated IL-6 levels (reference range: 0–124 pg/ml), 6/24 (25%) patients had increased IL-1β levels (reference range: 0–6.1 pg/ml) and 13/24 (54.2%) patients had elevated TNF-α concentration (reference range: 5.7–39.8 pg/mL). Among controls (*N* = 8/11), serum levels of IL-6 and IL1β were within the normal range for all analyzed subjects, while 3/8 (37.5%) controls showed TNF-α serum concentration above the laboratory reference range. When comparing TNF-α, IL-1β and IL-6 concentration between PANDAS patients and controls, no statistically significant difference was found between the two study groups (Table 2; Figure 1). In PANDAS patients, serum IL-17 levels ranged between 0.59 and 18.8 pg/ml (median [IQR]: 3.2 [2.22–6.08]), while in the control group between 0.64 and 9.6 (median [IQR]: 1.97 [0.82–3.07]). A trend toward higher IL-17 levels was detected in PANDAS patients compared to controls, although no statistical significance was found while comparing the study groups (Table 2; Figure 1). By correlating cytokine profile with clinical-anamnestic data and laboratory parameters within PANDAS group (i.e., age of disease onset, ASO titer, CRP, ESR, number of antibiotic courses in the year before our evaluation and neuropsychological tests score) a positive association was identified between ASO titer and serum TNF-α levels, and a negative association was identified between serum TNF-α levels and CY-BOCS score (Table 3; Figure 2). Yet, no statistically significant correlation was calculated (Table 3). The correlation between cytokine profile and other parameters (e.g., type of treatment, type of symptoms, etc.) is summarized in Supplementary Table A.1 (Supplementary Table A.1).

**Table 2:** Cytokine profile.

	Controls		Patients		
	Median	IQR	Median	IQR	*p*
TNF-α	22.47	19.66–54.30	37.98	21.52–130.02	0.651
IL-6	0	0–8.84	0	0–30.04	0.770
IL-1β	1	0–1.62	0.28	0–6.18	0.923
IL-17	1.97	0.82–3.07	3.2	2.22–6.08	0.471

IL, interleukin; IQR, interquartile range; *p*, *p*-value; TNF, tumor necrosis factor.

**Table 3:** Correlation between cytokine profile and clinical-anamnestic data and laboratory parameters in PANDAS patients.

		IL-17	IL-6	IL-1β	TNF-α
Age of onset	*r*	0.36	0.02	−0.11	0.08
	*p*	0.069	0.933	0.603	0.709
	*N*	26	24	24	24
ASO titer	*r*	0.10	0.21	−0.16	0.35
	*p*	0.628	0.336	0.450	0.095
	*N*	26	24	24	24
ESR	*r*	−0.28	−0.19	−0.29	−0.29
	*p*	0.170	0.373	0.180	0.173
	*N*	25	23	23	23
CRP	*r*	−0.24	0.06	−0.21	−0.13
	*p*	0.245	0.767	0.331	0.543
	*N*	26	24	24	24
N of antibiotic courses	*r*	0.04	−0.18	0.03	−0.20
	*p*	0.849	0.407	0.888	0.352
	*N*	26	24	24	24
CY-BOCS	*r*	−0.1	−0.2	−0.1	−0.4
	*p*	0.665	0.381	0.617	0.082
	*N*	25	23	23	23
YGTSS	*r*	0.17	0.08	0.23	−0.09
	*p*	0.414	0.710	0.297	0.684
	*N*	25	23	23	23

*r* = Pearson correlation coefficient, *p *= *p* value, *N* = number of subjects.

ASO, anti-streptolysin O; CRP, C-reactive protein; CY-BOCS, children's yale-brown obsessive-compulsive scale; ESR, erythrocyte sedimentation rate; YGTSS, yale global tic severity score.

## 4 Discussion

To better investigate the potential link between immunological profile and clinical phenotypes, both PANDAS patients and controls underwent a thorough phenotyping clinical evaluation and a structured collection of anamnestic data. Our study population showed a male:female (M:F) ratio of 3.33:1 confirming a higher prevalence of disease in males (4). All patients, at the time of our observation, presented with a relapsing-remitting course of disease in the previous year, in agreement with Gromark's proposed definition for PANS patients (10). Twenty-four patients had a prepubertal onset of disease while 2 patients developed symptoms at the age of 2, being considered early onset PANDAS variants as already described (13). Our PANDAS cohort included two pairs of siblings; moreover 3 patients had family history positive for rheumatic fever (first degree relatives) which could support the role of genetic susceptibility in the development of autoimmune sequelae following GABHS infection (23). Maternal history was positive for autoimmune disease in 14/24 (53.3%) PANDAS patients including 12/26 (46%) mothers diagnosed with autoimmune thyroiditis, confirming previously reported data (24). This evidence might suggest genetic predisposition to immune mediated diseases. The ASO titer was consistently higher than the reference value of our laboratory (i.e., >166 IU/ml) both in PANDAS patients and controls, being not significantly different between the two groups. This evidence suggests little significance of ASO titer in identifying PANDAS patients as already proposed by other authors (7, 25). Orefici et al., highlighted that a true GABHS infection might cause only a moderate increase in ASO titer which remains below the threshold considered the upper limit of normal titer (26). In agreement with the above-mentioned studies, we assume that ASO titer is not indicative itself to distinguish PANDAS patients and subjects with recurrent GABHS pharyngotonsillitis. It is, on the contrary, advisable to perform an accurate physical examination to exclude ongoing pharyngitis, and to directly identify the bacterium through pharyngeal swab, while retesting ASO titer to document its possible increase in time. This diagnostic process would potentially overcome the difficulty in establishing a causal relationship between streptococcal infection/exposure and the onset/recrudescence of OCD or tic symptoms, being one of the main critical aspects of PANDAS diagnosis. The clinical profile observed in our PANDAS cohort was characterized by a considerable incidence of neuropsychiatric symptoms other than OCD and tics (76.9% of patients), including emotional lability, depression, irritability, aggression, oppositional behaviors, behavioral developmental regression, deterioration in school performance (mostly in handwriting), separation anxiety and sleep disorder. Psychiatric, neurological and somatic comorbidities have been frequently described in PANS and PANDAS patients (4, 8, 27, 28), in agreement with our observations. Therefore, in our opinion, neuropsychiatric symptoms other than OCD and tics should be considered a principal feature of PANDAS disease rather than a comorbidity, thus supporting its clinical diagnosis. PANDAS pathophysiology is still a matter of debate. Several studies have been conducted to investigate possible immunological defects that might predispose to recurrent GABHS infections. Indeed, some authors hypothesized that patients presenting with PANDAS, TS or isolated OCD are more prone to infections due to possible immunological defects (29). Humoral immunodeficiency has been described in PANDAS patients when compared to healthy controls (30–32). More specifically, a study focused on IgA serum levels, showed significantly lower concentration of this immunoglobulin in PANDAS patients versus healthy age-matched subjects (31). The authors assumed that a compromised mucosal secretion of IgA might predispose to the development of recurrent tonsillitis and autoimmune disorders. However, none of the patients enrolled in the study fully expressed selective IgA deficiency (IgA < 7 mg/dl). Immunological assessment has been conducted in 431 PANS patients by Calaprice and colleagues: humoral immunodeficiency was reported in 25% of patients (low IgG levels in 46 patients, IgA deficiency and IgG subclass alterations were described) (23). However, the authors did not specify how many PANS patients responded to PANDAS criteria. Finally, Gamucci et al. proposed a diagnostic protocol for PANDAS/PANS that includes immunoglobulin profiling (IgM, IgG subclasses, and IgA) (25). Laboratory work-up including IgG, IgA, IgM and IgG subclasses has also been very recently proposed for PANS patients at disease onset (10). However, immunological studies conducted on PANDAS patients are mostly limited to single cases and immunological findings were not always compared with age matched reference intervals to rule out possible transitory immunodeficiency (due to age dependent maturation of the immune system). Furthermore, defective antibody production should always be investigated when the total immunoglobulin level is only slightly decreased in the setting of recurrent bacterial infections (33). We therefore performed a comprehensive screening of antibody-mediated immune function, by measuring immunoglobulin levels, IgG subclass levels and specific vaccine induced antibody production. In addition, complete blood count and flow cytometry analysis were able to quantify T-B and NK cells. Our patients did not display defects of the immune system potentially responsible for recurrence of GABHS infections. In addition, when comparing immunological findings between the two study groups, no statistically significant difference was found. One patient presented with incomplete isolated IgA deficiency, being considered a normal finding in prepubertal age. Literature data described so far are not confirmed by our observations which do not support the recommendations of a diagnostic protocol for PANDAS/PANS including humoral and cellular immune response analysis. Notably, 42.3% of PANDAS patients showed reduced C3 and C4 levels. Complement analysis was limited to C3–C4 components, as defects in C3 produce a clinical phenotype that might be indistinguishable from humoral defects, although much less frequent. Further studies are needed to establish the potential role of complement, either due to deficiency or consumption, in PANDAS pathophysiology by also assessing CH50. Specific cytokines have been analyzed in PANDAS patients on different tissues to investigate a possible inflammatory status and to unravel potential disease biomarkers. However, a limited number of heterogeneous studies is to date available with conflicting results. Cytokine profile, assessed in 12 PANDAS patients on tonsillar tissue, showed higher TNF-α and eotaxin-3 expression and lower CXCL8, CXCL10, IL-17A, IFN-γ, IL-10 and IL-12 expression compared to controls (34). Nonetheless, the authors suggested further investigations finalized to a specific interpretation of reduced levels of IL-17 and IFNγ (not compatible with the presumptive autoimmune mechanism of PANDAS disease). A panel of proinflammatory cytokines has also been analyzed in a PANDAS cohort on CSF and compared to healthy controls, without showing statistically significant difference between the two study groups (35). Only a few studies have been conducted to analyze serum cytokines concentration in PANDAS patients. When analyzing a PANDAS cohort, Singer et al. did not find significant difference between patients and controls for IL-4, IL-10, IL-12, and TNF-α serum levels during both remitting phases and exacerbations (36). Leckman et al. analyzed serum concentration of 9 cytokines in 11 PANDAS patients (being a subgroup of 46 patients presenting with OCD + tics) during 24 months by collecting serum specimens every 4 months and during exacerbations (37). At baseline PANDAS patients and controls did not show any significant difference in cytokine profile while, during exacerbations, an increase in IL-12 and TNF-α was detected in all patients compared to controls. In another study, a panel of 9 cytokines was assessed in 21 children with chronic tic disorder, including 5 PANDAS patients. The authors, similarly, to Leckman et al., reported that 77% of patients expressed significantly higher levels of serum TNF-α during tic exacerbations (38). Recently, higher TNF-α concentration has been reported in a symptomatic PANS cohort with chronic course of disease (10). Both above-mentioned studies, despite not aimed at studying exclusively PANDAS patients, highlighted higher levels of TNF-α during disease exacerbations. In agreement with this evidence, when analyzing our PANDAS cohort, more than half of patients (54.2%) showed high serum concentrations of TNF-α. Although TNF-α concentration did not statistically differ between the study groups, we hypothesize that a larger study population could reach statistical significance. These findings are undoubtedly of interest, as it is known that increased levels of TNF-α play a role in promoting BBB permeability, enabling autoimmune response against CNS and could therefore contribute to PANDAS pathophysiology (39). We hypothesized that PANDAS patients exhibit a status of enduring systemic inflammation, being characterized by excessive and chronic production of inflammatory cytokines, justifying the persistence of neuropsychiatric symptoms regardless of the phase of the disease and beyond infectious episodes. Our patients showed high levels of ASO titer, inflammatory cytokines, and neuropsychological tests scores in the absence of an ongoing infection and with negative routine infection/inflammatory markers (CRP and ESR). In order to investigate the potential correlation between inflammatory status and the severity of clinical phenotype of PANDAS patients, we compared cytokines concentration to clinical and laboratory parameters. TNF-α was found to be inversely correlated with OCD severity measured by the CY-BOCS (*r* = −0.4; *p* = 0.082). A similar trend for an inverse correlation between TNF-α plasma levels and CY-BOCS was described in children with TS by Gabbay et al. (39). The authors did not further explain these results. Additional studies with larger sample size are needed to further investigate the notion of these findings. Our cytokine profiling included IL-17 detection in serum of both PANDAS patients and controls. To the best of our knowledge, surprisingly, this cytokine has never been studied in PANDAS patients’ serum, despite its possible role in PANDAS pathophysiology has been suggested in few preclinical studies above-mentioned (11, 12). Moreover, a general mechanism by which infectious agents inducing Th17 immunity can trigger CNS autoimmune diseases has been often suggested and a consistent body of evidence suggests that Th17 and IL-17A intervene in the development of many CNS diseases (40). Some authors assumed that even in PANDAS patients, high serum levels of IL-17 could reflect higher levels of IL-17 in the brain, as happens with other CNS autoimmune conditions (41). In agreement with this hypothesis, we observed a trend toward higher IL-17 levels in the PANDAS patients compared to the control group. These results strongly encourage further investigation of the role of IL-17 in a larger number of PANDAS patients. Indeed, the potential role of IL-17 serum concentration as peripheral specific biomarker for disease progression and treatment response would be of significant value, being this analysis easily accessible and not requiring invasive procedure.

## 5 Study limitations

The main limitation of our study is represented by a small sample size, which may result in incomplete statistical representation of our data. Indeed, a trend toward higher TNF-alpha and IL-17 levels, and lower C3 levels, was detected in the PANDAS patients compared to the control group. This data should be explored in further studies with larger sample size and appropriate statistical correlations accounting for age, gender, and current medications. It would also be valuable to perform longitudinal immunological assessments measured during flares and after specific therapies. Th17 analysis and total complement activity would also be strongly recommended. Due to the potential influence of biological and technical variables on cytokine immunoassay interpretation, we maintained consistency by utilizing the same method, platform, and laboratory when comparing cytokine levels between the two groups. Future studies would benefit from performing repeated measurements of cytokine concentrations over time.

## 6 Conclusions

Our results suggest that PANDAS patients may have higher TNF-α and IL-17 serum concentration compared to controls, potentially representing a state of persistent systemic inflammation due to an enhanced immune response to GABHS. These data failed to reach statistical significance; therefore, future investigative efforts should focus on reproducing these important findings in a larger population to confirm this hypothesis. To the best of our knowledge, this study is the first to analyze IL-17 serum concentration in PANDAS patients and our results encourage further studies to confirm the potential role of IL-17 and TNF-α as specific peripheral biological markers in PANDAS. Finally, our observations highlight the limits of ASO titer in supporting PANDAS diagnosis, being unable to prove the causal association with GABHS infections. To overcome the difficulty to correlate PANDAS onset to GABHS infection further studies should always consider at least two antibodies [ASO and antideoxyribonuclease B (anti-DNaseB)] together with a pharyngeal swab for GABHS culture for the diagnosis of new infection. Conversely, the potential value of neuropsychiatric symptoms other than OCD and tics in PANDAS diagnosis has been suggested.

## Data Availability

The raw data supporting the conclusions of this article will be made available by the authors, without undue reservation.
