# Supplementary material for: Immunological characterization of an Italian PANDAS cohort
Source: Front Pediatr. 2024 Jan 4;11:1216282. doi: 10.3389/fped.2023.1216282 (PMC10794562; doi:10.3389/fped.2023.1216282)
Supplement: Supplementary file 1 [file Table1.docx]

Supplementary Material

**Title:**Immunological Characterization of an Italian PANDAS Cohort

Authors:
Lucia Leonardi1*, Giulia Lorenzetti2, Rita Carsetti3, Eva Piano Mortari3, Cristiana Alessia Guido1, Anna Maria Zicari1, Elisabeth Förster-Waldl4, Lorenzo Loffredo5, Marzia Duse1, Alberto Spalice1

*** Correspondence:** Lucia Leonardi: lucialeonardi@yahoo.it; lucia.leonardi@uniroma1.it

**Affiliations:**^1^Department of Maternal, Infantile, and Urological Sciences, Faculty of Medicine and Dentistry, Sapienza University of Rome, Italy
^2^Department of Pediatrics, University of Rome Tor Vergata, Italy
^3^B Cell Physiopathology Unit, Immunology Research Area, Bambino Gesù Children's Hospital (IRCCS), Italy
^4^Medical University of Vienna, Department of Pediatrics & Adolescent Medicine, Division of Neonatology, Pediatric Intensive Care and Neuropediatrics Center for Congenital Immunodeficiencies
^5^Department of Clinical, Internal Medicine, Anesthesiologic and Cardiovascular Sciences, Sapienza University of Rome, Italy

## Supplementary Table A.1. Cytokines And Clinical-Therapeutic Correlation

|  |  |  | *IL-17* |  | *IL-6* |  | *IL-1β* |  | *TNF-α* |  |
| --- | --- | --- | --- | --- | --- | --- | --- | --- | --- | --- |
|  |  | *N* | *Median* | *IQR* | *Median* | *IQR* | *Median* | *IQR* | *Median* | *IQR* |
| **Penicillin** | No | 19 | 3.2 | 2.1-4.3 | 0 | 0-33.2 | 0.6 | 0.-4.1 | 51 | 24.2-152.7 |
|  | Yes | 7 | 3.2 | 2.7-8.4 | 3 | 0-37.4 | 0 | 0-14.1 | 23.3 | 20.4-89.5 |
|  | *p* |  | 0.333 |  | 0.467 |  | 0.584 |  | 0.205 |  |
| **Amoxicillin/ clavulanate** | No | 7 | 3.2 | 2.3-3.2 | 0.89 | 0-39.9 | 0.08 | 0-2.5 | 24.8 | 20.0-146.4 |
|  | Yes | 19 | 3.2 | 2.1-6.2 | 0 | 0-20.0 | 0.28 | 0-8.3 | 43.8 | 23.8-100.6 |
|  | *p* |  | 0.598 |  | 0.604 |  | 0.434 |  | 0.505 |  |
| **Azithromycin** | No | 18 | 3.2 | 2.6-6.4 | 0.5 | 0-36.7 | 0.28 | 0-8.6 | 38 | 21.1-154.6 |
|  | Yes | 8 | 2.3 | 0.8-3.2 | 0 | 0-23.4 | 0.7 | 0.02-2.21 | 44.8 | 22.9-128.7 |
|  | *p* |  | 0.063 |  | 0.463 |  | 0.95 |  | 0.854 |  |
| **Cognitive-behavioral therapy** | No | 22 | 3.2 | 2.1-6.1 | 0.45 | 0-37.6 | 0.57 | 0-6.2 | 51.2 | 24.9-144.5 |
|  | Yes | 4 | 3.2 | 2.5-14.8 | 0 | 0-20.5 | 0 | 0-7.9 | 20.8 | 20.1-82.7 |
|  | *p* |  | 0.564 |  | 0.353 |  | 0.34 |  | 0.121 |  |
| **Tonsillectomy** | No | 23 | 3.2 | 2.3-6.2 | 0 | 0-21.8 | 0.28 | 0-2.7 | 32.2 | 21.8-142.6 |
|  | Yes | 3 | 2.69 | 1.01-3.2 | 27.4 | 0.95-67.4 | 10.6 | 0-24.7 | 85 | 18.2-103.3 |
|  | *p* |  | 0.329 |  | 0.096 |  | 0.193 |  | 0.896 |  |
| **Type of symptoms** | Tics | 11 | 3.2 | 2.3-8.4 | 5.1 | 0-30.9 | 0.28 | 0-9.1 | 85 | 25.3-146.4 |
|  | OCD | 2 | 10.5 | 2.3-18.7 | 0 | 0-0 | 0 | 0-0 | 20.8 | 20.5-21.1 |
|  | OCD + tics | 13 | 3.2 | 1.6-37 | 0 | 0-39.9 | 0.85 | 0.08-7.24 | 31.1 | 22.3-138.9 |
|  | *p* |  | 0.593 |  | 0.46 |  | 0.252 |  | 0.16 |  |
| **Other neuropsychiatric**  **symptoms** | No | 7 | 6.04 | 2.4-8.4 | 0.89 | 0-12.7 | 0 | 0-2.5 | 26.8 | 24.8-146.4 |
|  | Yes | 19 | 3.2 | 2.0-3.2 | 0 | 0-40.0 | 0.85 | 0-8.3 | 43.8 | 20.8-121.1 |
|  | *p* |  | 0.143 |  | 0.972 |  | 0.171 |  | 0.68 |  |
| **Symptoms in the**  **previous months** | No | 12 | 3.2 | 2.0-4.0 | 0 | 0-10.8 | 0.18 | 0-1.20 | 31.6 | 21.7-80.0 |
|  | Yes | 14 | 3.2 | 2.2-7.7 | 0.92 | 0-37.6 | 1.94 | 0-8.7 | 78.3 | 21.5-165.4 |
|  | *p* |  | 0.465 |  | 0.257 |  | 0.299 |  | 0.453 |  |

IQR, interquartile range; OCD, obsessive-compulsive disorder; *p*, *p*-value.
